# Constructing a Carbon-Encapsulated Carbon Composite Material with Hierarchically Porous Architectures for Efficient Capacitive Storage in Organic Supercapacitors

**DOI:** 10.3390/ijms23126774

**Published:** 2022-06-17

**Authors:** Rene Mary Amirtha, Hao-Huan Hsu, Mohamed M. Abdelaal, Ammaiyappan Anbunathan, Saad G. Mohamed, Chun-Chen Yang, Tai-Feng Hung

**Affiliations:** 1 Battery Research Center of Green Energy, Ming Chi University of Technology, 84 Gungjuan Rd., Taishan Dist., New Taipei City 24301, Taiwan; reneamirthasdj@gmail.com (R.M.A.); u08137122@mail2.mcut.edu.tw (H.-H.H.); mohamedbec@yahoo.com (M.M.A.); ammaiyappananbunathan1@gmail.com (A.A.); ccyang@mail.mcut.edu.tw (C.-C.Y.); 2 Tabbin Institute for Metallurgical Studies (TIMS), Tabbin, Helwan 109, Cairo 11421, Egypt; sgmmohamed@gmail.com; 3 Department of Chemical Engineering, Ming Chi University of Technology, 84 Gungjuan Rd., Taishan Dist., New Taipei City 24301, Taiwan; 4 Department of Chemical and Materials Engineering, Chang Gung University, 259 Wenhua 1st Rd., Guishan Dist., Taoyuan 33302, Taiwan

**Keywords:** supercapacitors, hierarchical porous activated carbon, hydrogel, composite materials, clean energy technology

## Abstract

Hierarchical porous activated carbon (HPAC) materials with fascinating porous features are favored for their function as active materials for supercapacitors. However, achieving high mass-loading of the HPAC electrodes remains challenging. Inspired by the concepts of carbon/carbon (C/C) composites and hydrogels, a novel hydrogel-derived HPAC (H-HPAC) encapsulated H-HPAC (H@H) composite material was successfully synthesized in this study. In comparison with the original H-HPAC, it is noticed that the specific surface area and pore parameters of the resulting H@H are observably decreased, while the proportions of nitrogen species are dramatically enhanced. The free-standing and flexible H@H electrodes with a mass-loading of 7.5 mg/cm^2^ are further prepared for electrochemical measurements. The experiments revealed remarkable reversible capacitance (118.6 F/g at 1 mA/cm^2^), rate capability (73.9 F/g at 10 mA/cm^2^), and cycling stability (76.6% of retention after 30,000 cycles at 5 mA) are delivered by the coin-type symmetric cells. The cycling stability is even better than that of the H-HPAC electrode. Consequently, the findings of the present study suggest that the nature of the HPAC surface is a significant factor affecting the corresponding capacitive performances.

## 1. Introduction

Electrochemical-based energy storage devices such as metal–ion batteries/capacitors and supercapacitors are recognized as alternative choices for electricity storage owing to their high flexibility, remarkable reversibility, and simple maintenance as compared to other electric storage technologies [1,2]. Recently, preparing electrodes with high-mass loading has attracted much attention because the active material ratio in devices should be increased as much as possible to provide high total capacitances and gravimetric or volumetric energy densities [3,4,5]. However, challenges associated with this target still remain, especially in employing the hierarchical porous activated carbon (HPAC) as the active material for supercapacitors. This can be attributed to its huge specific surface area (typically more than 1000 m^2^/g), leading to the limited mass loading (normally 1 to 2 mg/cm^2^ by a doctor blade method) [6]. Therefore, developing an HPAC that retains the distinctive textural properties and increases the mass loading of the resulting electrode is of interest and highly desirable [3,4,7,8,9]. 

Carbon/carbon (C/C) composites are demonstrated to possess a variety of characteristics, i.e., high specific strength, remarkable electrical and thermal conductivities, and excellent dimensional stability [10]. Given the diverse properties, they are beneficial in the field of biomedical, automobile industries, and aeronautics. To realize the C/C composites, it is reported that polymer infiltration pyrolysis and chemical vapor infiltration were commonly adapted [11,12]. As a result, compact and dense C/C composites were obtained, particularly from the repeatedly manufacturing processes. Such a configuration would be beneficial to enhance the mass loading, but not favorable in terms of electrolyte penetration and ionic transportation.

To maximize the electrolyte storage and ionic conductivity within a high mass loading electrode, the hierarchical porosities including micro-, meso-, and macro-pores are crucial. For example, the charges are primarily adsorbed/desorbed inside the micropores. As for the latter two, they can contribute to (i) an electrolyte reservoir, (ii) enlarging the ionic diffusion rate, and (iii) facilitating the migration of large ions/molecules [13,14,15,16]. Undoubtedly, utilizing a template and activation are straightforward approaches for synthesizing the HPAC [14,17]. Even so, it will be highly appreciated if the greener templates and activators were chosen owing to resolve environmental issues and promote cost-effectiveness issues [9,18,19,20,21].

In our recent study, hydrogel-derived HPAC (H-HPAC) synthesized by pyrolysis of polyvinylpyrrolidone hydrogel under an argon atmosphere at 900 °C was successfully obtained [9]. The merits of H-HPAC can be attributed to (i) numerous water molecules encapsulated within PVP hydrogel efficiently serving as green templates, and (ii) the simultaneous function of K_2_CO_3_ as an initiator for hydrogel formation and an activator to enable rich porous conformations. Accordingly, the resultant H-HPAC revealed fascinating structural features and distinguished capacitive performances for electrochemical storage applications. Inspired by the concepts of C/C composites and hydrogels, an alternative H-HPAC encapsulated H-HPAC (H@H) composite material was proposed in the present study. After systemically investigating the physicochemical and morphological properties, the H@H electrodes with a mass loading of 7.5 mg/cm^2^ were prepared to evaluate the electrochemical performances of supercapacitors that were assembled with an organic electrolyte. Moreover, various factors such as the physicochemical and textural properties that affect the corresponding electrochemical performances were also explored. On the basis of the results and viewpoints reported here, it is reasonably anticipated that such a strategy also has general applicability to other C/C composites.

## 2. Results and Discussion

### 2.1. Characterizations of Hydrogel-Derived Hierarchical Porous Activated Carbon (H-HPAC)-Encapsulated H-HPAC (H@H) Composite Material

**Figure 1:**
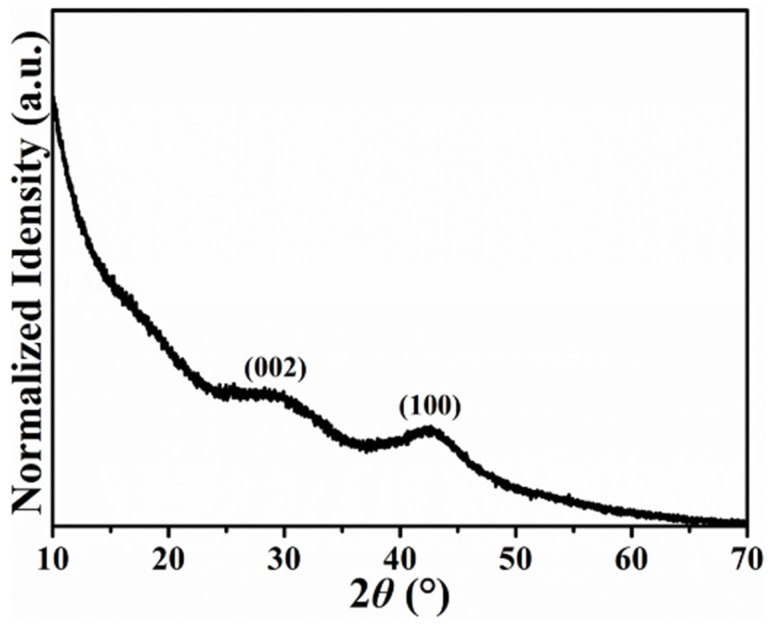
Normalized PXRD pattern of H@H composite material.

To explore the physicochemical and morphological properties of H@H composite material, it was systematically investigated by PXRD, Raman, SEM, TEM, BET, EA, and XPS, with the corresponding characteristics compared with those of H-HPAC. Figure 1 depicts the normalized PXRD pattern of H@H to show its crystalline structure. As can be seen, only two broad peaks, assigned to the (002) and (100) planes of carbon (JCPDS No.: 41-1487), were reflected, which was consistent with the original H-HPAC and other activated carbon materials [9,14,17,20,22]. In line with the possible formation mechanism for PVP hydrogel proposed in our previous study, the cross-linking reactions among the polymer chains were initiated by the coordination between potassium cations (K^+^) and oxygen anions (O^−^) [9]. When the K_2_CO_3_ solution was added to the PVP/H-HPAC solution, it is reasonably postulated that the K^+^ would also interact with the H-HPAC because 4.7% of the oxygen present in the original H-HPAC was verified by elemental analysis [9]. If so, the K^+^ coordination among the H-HPAC would be covered by the PVP hydrogel. Even so, no peaks associated with unreacted K_2_CO_3_ were observed from the PXRD pattern, implying that the purity of H@H was not affected by the presence of H-HPAC after thoroughly rinsing with DI water.

**Figure 2:**
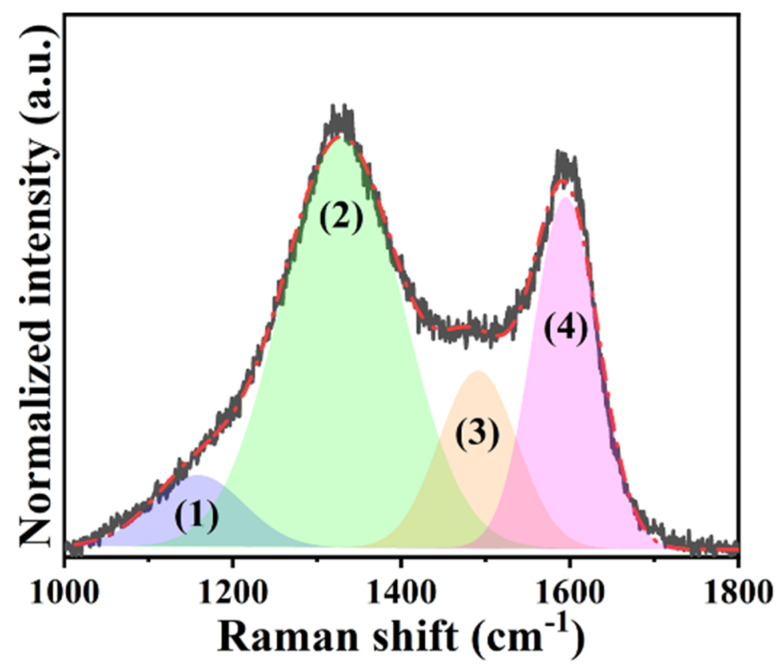
Fitted Raman spectrum of H@H composite material.

Raman characterization is another approach that can directly examine the crystallinity of carbonaceous materials. The normalized Raman spectrum illustrated in Figure 2 shows two distinct peaks representing the D (~1327 cm^−1^) and G (~1593 cm^−1^) bands. Besides, it is meaningful to discuss the intensity ratio between D and G bands (*I*_D_/*I*_G_) because the degree of defects within the carbonaceous materials can be further evaluated. The value calculated from the H@H was 1.16, the same as the H-HPAC and close to that of the A-PVP-NC (1.18) [21]. The high *I*_D_/*I*_G_ ratio suggests that many defects and/or highly disordered degrees exist, as is generally observed in the carbonaceous materials with numerous functional groups [23,24]. Consequently, we could ascribe this result to the presence of heteroatoms (i.e., N and O) and lower crystallinity, as demonstrated in the original H-HPAC [9]. On the other hand, the corresponding Raman spectrum was sequentially deconvoluted into four peaks (labeled peaks (1)–(4)) since the integrated area ratio of sp^3^ to sp^2^ (*A*_sp_^3^/*A*_sp_^2^) has been shown to provide helpful information on the nature of carbon, e.g., a low *A*_sp_^3^/*A*_sp_^2^ ratio indicates that a large amount of carbon exists as the sp^2^ type [25,26]. Among them, peaks (2) and (4) are associated with sp^2^-type carbon, whereas the others are related to sp^3^-type carbon [27]. The integrated area ratio of sp^3^ to sp^2^ (*A*_sp_^3^ / *A*_sp_^2^) was calculated to be 0.28, which was identical to that of H-HPAC [9]. This result signifies that the H@H still retained a high proportion of sp^2^-type carbons, even with intrinsically lower crystallinity.

**Figure 3:**
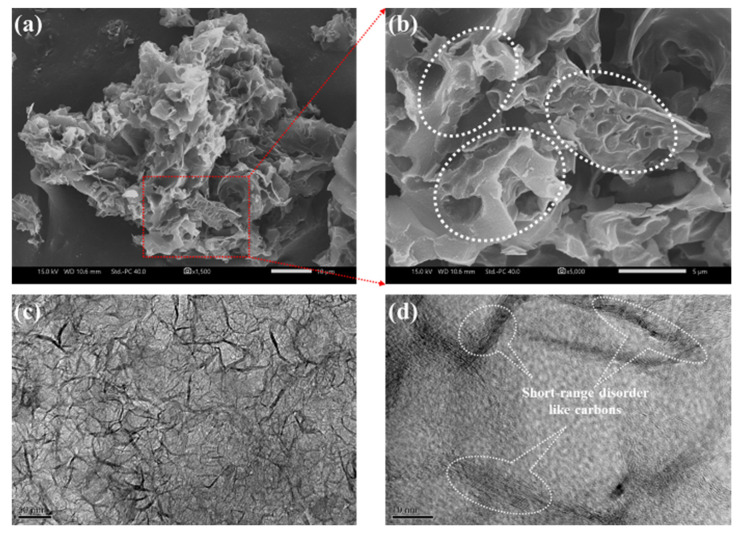
(**a**,**b**) SEM and (**c**,**d**) TEM micrographs of the H@H composite material. Scale bar: (**a**) 10 μm, (**b**) 5 μm, (**c**) 50 nm, and (**d**) 10 nm.

To examine the morphological features of H@H, micrographs were captured using SEM and TEM. The hierarchically porous architectures constructed by interconnected carbonaceous frameworks were clearly visible from the low-magnification SEM micrographs in Figure 3a,b. It is worth mentioning that rough surfaces with numerous voids were found, as indicated by white circles in Figure 3b. The diverse porous configurations are reasonably attributed to the water molecules encapsulating within PVP/H-HPAC hydrogel being evaporated and the activation process by interacting the carbonized residues with K_2_CO_3_ under 900 °C (i.e., K_2_CO_3_ + 2C → 2K + 3CO, K_2_CO_3_ → K_2_O + CO_2_, C + CO_2_ → 2CO) [18,19]. Based on the morphologies found in SEM, it is expected that similar characteristics were also exhibited, as shown in Figure S2 and Figure 3c at different TEM magnifications. Moreover, it is seen that the short-range disorders, such as carbon lattices, highlighted by white circles were displayed in Figure 3d, which might be correlated with skeleton collapse after high-temperature pyrolysis [28].

**Figure 4:**
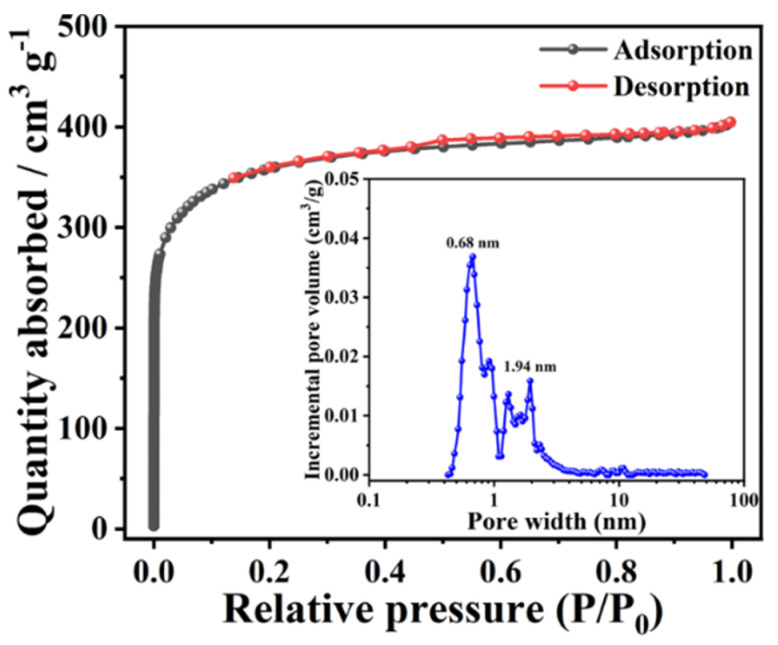
Nitrogen adsorption–desorption isotherm of H@H composite material collected by an accelerated surface area and porosimetry system at 77 K. Inset shows the pore size distribution curve determined by the 2D-NLDFT model.

Given the positive results found in SEM and TEM, it is believed that the textural characteristics of H@H would not be significantly affected. To accurately classify pores and determine the specific surface area (SSA), the nitrogen adsorption–desorption measurement was conducted, and the corresponding isotherm is shown in Figure 4. As plotted, not only a high volume of nitrogen gases were adsorbed and desorbed at low relative pressure (i.e., Type I isotherm), but also the predominant pore diameter was less than 2 nm (inset of Figure 4), confirming the microporous feature for H@H [9,21,29]. However, all values diminished except for the pore size distributions of ultramicropores as compared with H-HPAC (see Table 1). For instance, the SSA value determined by the Brunauer–Emmett–Teller (BET) method decreased by about 35%. Accordingly, the SSA values contributed by micropores and mesopores were decrease to 1246 m^2^/g and 45 m^2^/g, respectively. In particularly, the latter was reduced by even approximately 84%. When preparing the PVP/H-HPAC composite hydrogel, these pores within the H-HPAC would be filled with the viscous PVP solution so that the PVP blocked the original pores after drying of the composite hydrogel at 120 °C. In addition to the issue mentioned above, the decrease in the textural parameters might also be actuated by the possible interaction between H-HPAC and K^+^. Such a phenomenon is rationally postulated to affect the cross-linking degree of the PVP/H-HPAC composite hydrogel, as shown by the XRD characterization, leading to fewer sites for activation.

The compositional information and chemical environments of H@H were identified through EA and XPS, respectively. As quantified by the former, the proportions of carbon, nitrogen, and oxygen in the as-synthesized H@H were 76.2%, 0.58%, and 4.5%, respectively. In comparison with the original H-HPAC, the carbon content was decreased (76.2% vs. 95.1%), but the nitrogen species was enhanced (0.58% vs. 0.23%), while the oxygen species was similar (4.5% vs. 4.7%). It would be attributed the variation in carbon to the cross-linking degree of PVP/H-HPAC composite hydrogel. As for the increase in the nitrogen species, the following possible reasons could be given. It is reported that the nitrogen species included in the carbon precursor/char were preferentially removed during chemical activation with K-based salts [20]. However, as previously mentioned in XRD characterization, K^+^ would also interact with the original H-HPAC due to the presence of oxygen, reducing the concentration of K^+^ that was coordinated to the PVP as compared to the preparation of the original H-HPAC. According to Ref. [20] and our experimental results [9,21], the higher nitrogen percentage in the H@H could be attributed to the lower interaction between the K^+^ and carbonaceous residues.

**Figure 5:**
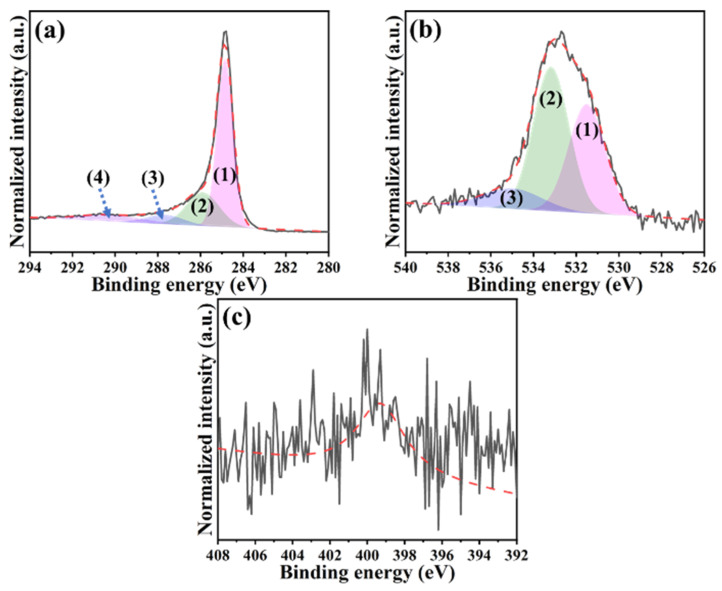
High-resolution XPS spectra of H@H composite material: (**a**) C 1s ((1) for C=C, (2) for C−O, (3) for C=O, and (4) for O=C−O bonds), (**b**) O 1s ((1) O=C−O, (2) C=O, and (3) C−O bonds), and (**c**) N 1s.

Figure 5 provides the high-resolution XPS spectra that were analyzed using the Gaussian–Lorentzian fitting method. From the EA result, the presence of nitrogen atoms within the H@H was already demonstrated. Therefore, the C-N bonding in the C 1s spectrum did not particularly point out for better reading. As revealed in Figure 5a, the C 1s spectrum was deconvoluted into four peaks: (1) C=C bond at 284.8 eV, (2) C-O bond at 285.9 eV, (3) C=O bond at 287.8 eV, and (4) O=C-O bond at 290.2 eV [30,31]. It is known that the first peak was assigned to the sp^2^-type carbon, while the rest corresponded to the contribution of sp^3^-type carbon [27]. As for the O 1s spectrum (Figure 5b), three peaks fitted at 531.5 eV, 533.1 eV, and 535.0 eV have appeared, representing (1) O=C-O, (2) C=O, and (3) C-O bonds, respectively [32]. Even though lower nitrogen content was shown in the EA results, the N 1s peak in the binding energy between 396 eV and 402 eV still can be detected (Figure 5c) [9,21,33].

Based on the results discussed in this section, the as-prepared H@H produced from thermal pyrolysis of the PVP/H-HPAC composite hydrogel combines various benefits, such as good purity, hierarchical porous characteristics, and high proportions of sp^2^-type carbons, as well as nitrogen species. Although the structural parameters were significantly altered, it is of interest to consider the influence of physicochemical and textural features of the H@H on the corresponding electrochemical performance as organic supercapacitors.

### 2.2. Electrochemical Performances of H@H in Symmetric Supercapacitor

**Figure 6:**
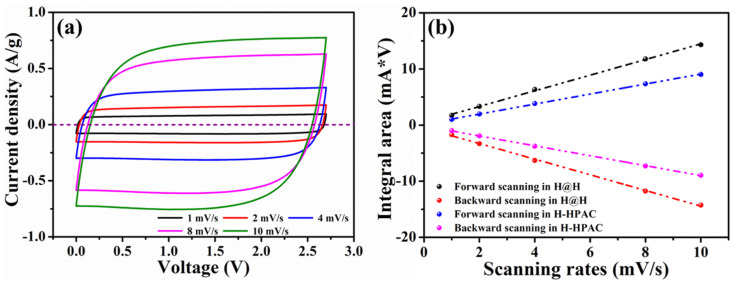
(**a**) Cyclic voltammograms collected in the voltage range between 0 and 2.7 V at scanning rates from 1 to 10 mV/s and (**b**) dependence of the integral area on scanning rate, with the values acquired from (**a**) and Ref. [9] for H@H and H-HPAC, respectively.

To evaluate the capacitive efficiencies of the H@H electrode, coin-type symmetric cells were fabricated to conduct the cyclic voltammetry (CV) and galvanostatic charge-discharge (GCD) measurements in the voltage window between 0 V and 2.7 V by different scanning rates and current densities. Although the SSA values and relative textural parameters were less than the H-HPAC, as discussed previously, the typical curves in nearly rectangular shapes with good symmetries were reflected from the H@H (Figure 6a), despite gradually increasing the scanning rate to 10 mV/s. During CV cycling, the integral area from the cyclic voltammogram is associated with the charges adsorbed and desorbed among the active materials. Figure 6b compares the integral area of H@H and H-HPAC acquired in terms of the forward and backward scanning. As can be seen, the values linearly increased with the scanning rate. In addition, the values for the H@H electrode were superior those for the H-HPAC electrode; this was correlated with the different mass loading (7.5 mg/cm^2^ vs. 5.1 mg/cm^2^). On the other hand, the voltage delay (ΔV) is regarded as an important indicator providing similar information to the IR drop. To discuss this discrepancy, we consider the voltage that reached zero for a current density at 1 mV/s as a reference. The ΔV values were then calculated by comparing the difference between the voltage recorded from each scanning rate and the reference; the corresponding data are visible in Figure S3. Under the voltage range of 0 V to 2.7 V and a scanning rate of 1 mV/s, the reference data for each free-standing electrode were 45 mV (HPAC electrode: 230 µm [7]), 22 mV (H@H electrode: 160 µm) and 12 mV (H-HPAC electrode: 100 µm [9]), respectively. With increase in scanning rate to 10 mV/s, it was found that the ΔV value was 128 mV, i.e., a 64% increase as compared to that of the H-HPAC (78 mV) [9]. This could be ascribed to the thickness of the H@H electrode, which was ~60% more than the H-HPAC electrode, prolonging the pathway for electron transportation. 

**Figure 7:**
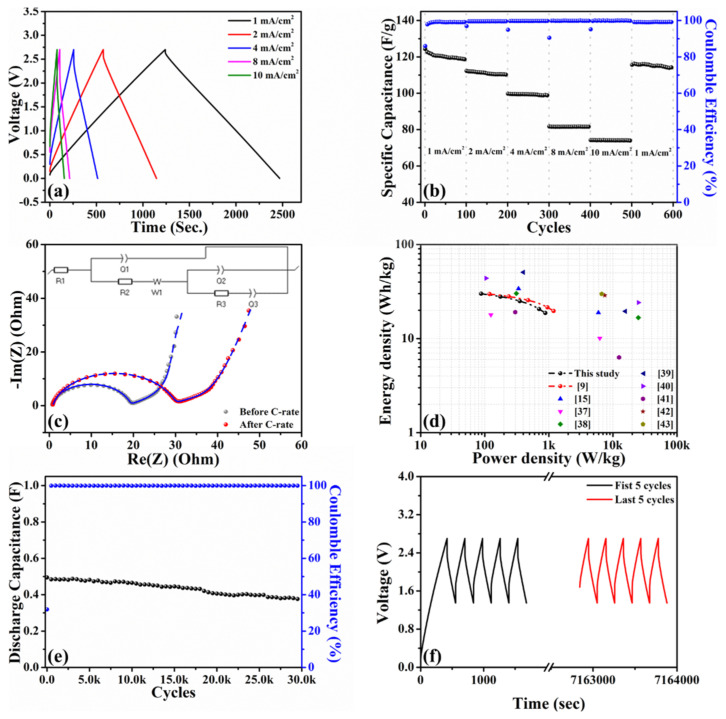
(**a**) Galvanostatic charge–discharge profiles, (**b**) rate capabilities, (**c**) electrochemical impedance spectra, (**d**) Ragone plots, (**e**) discharge capacitance as a function of cycle number of the H@H electrode in a coin-type symmetric cell, and (**f**) galvanostatic charge–discharge profiles of the first and last five cycles received from (**e**). The inset of (**c**) illustrates the equivalent circuit model used for the parameter fitting [45].

Figure 7a presents the GCD profiles measured using the same voltage window as in the CV test but with current densities from 1 mA/cm^2^ to 10 mA/cm^2^. Contributing to the ideal electric double-layer behavior and high reversibility, as shown in Figure 6a, the linear and symmetric charge–discharge behavior at each current density was observed. The specific capacitance discharged from the H@H electrode at the 100th cycle was 118.6 F/g at 1 mA/cm^2^ with 99% Coulombic efficiency. This result was slightly higher than that outputted from the H-HPAC electrode (117.5 F/g [9]), implying that the specific capacitance was not appreciably affected by the thickness when applying a small current density. Additionally, the stable discharge capacitances of 110.2, 98.8, 81.6, and 73.9 F/g are compared in Figure 7b. The tendency for capacitance decay was the same as the H-HPAC, but the values were observably declined while the current densities were above 4 mA/cm^2^, which could also be attributed to the change in thickness. However, 96.4% of the recovery in capacitance after 100 cycles was obtained when the current density was returned to 1 mA/cm^2^. Figure 7c shows the EIS spectra that were recorded before and after rate-capability testing. It is recognized that charge transfer resistance (*R*_ct_) includes ionic and electronic resistances. The former is the resistance to the mobility of ionic electrolytes inside the textual pores of the electrode, while the latter comprises the intrinsic resistance of the electrode material and the contact resistance between the active layer and the current collector [34]. For the rate-capability testing, the same electrode conditions (i.e., composition, working area, and thickness) were used, based on the hypothesis that the electronic resistance should be no significant differences. Following repeated charging–discharging processes, the *R*_ct_ value increased from 18.5 Ohm to 29.1 Ohm. As reported, the diameter of solvated ions for TEA^+^ and BF_4_^−^ are 1.35 nm and 1.40 nm, respectively [35]. Hence, the bulky solvated ions would accumulate within the pores of H@H, further blocking the ionic transport and causing an increase in *R*_ct_ as well as capacitance decay [36].

The energy and power densities calculated from the data presented in Figure 7b and the equations shown in Section 3.4 are plotted in Figure 7d. As indicated, the values ranged from 30.0 Wh/kg (@ 1 mA/cm^2^) to 18.7 Wh/kg (@ 10 mA/cm^2^) for the former, and from 88.1 W/kg (@ 1 mA/cm^2^) to 881.7 W/kg (@ 10 mA/cm^2^) for the latter. To compare with the H-HPAC electrode, ~74% of the power density was outputted by the H@H electrode for all current densities. On the basis of the same electrolyte and similar voltage window, the H@H electrode exhibits reasonable energy and power densities, comparable with the results reported previously (Figure 7d) [9,15,37,38,39,40,41,42,43]. Considering the cycling stability, the accelerated experiment conducted in the voltage ranged from 1.35 to 2.7 V (i.e., 50% of the state of discharge) and the current of 5 mA was used; the corresponding result is displayed in Figure 7e. The initial capacitance discharge to 1.35 V was ~0.5 F. After 30,000 cycles, about 76.6% of capacitance retention and ≥99.5% of Coulombic efficiency were found, respectively. Although the *R*_ct_ value increased from 32.8 Ohm to 45.1 Ohm (see Figure S4), the H@H electrode provided a better lifespan than that of the H-HPAC electrode (capacitance retention: 76% after 10,000 cycles) [9]. It is reported that increasing the mass loading or electrode thickness leads to a decrease in capacitance and the rate capability of the electrode materials, which is related to the decreased accessible surface area, increased electrical resistance, prolonged ion transport channels, and poor electrolyte wetting [4]. Besides, the variety of heteroatom dopants and their corresponding amounts, as well as the porosity characteristics, within HPAC were also significant influences [17,44]. The comparison of the electrochemical performance of H@H and that reported for HPAC in symmetric supercapacitors using 1 M TEABF_4_/PC electrolyte is listed in Table S1. The variations in the electrochemical performance of H@H can be attributed to the following. First, even though the SSA value and pore parameters were lower than those for H-HPAC, the increased thickness of the H@H electrode would compensate for their active sites of capacitive storage, because the specific capacitance generated from the H@H electrode at 1 mA/cm^2^ was slightly enhanced. Second, the number of nitrogen species doped in the H@H was increased by up to 152% in comparison with the H-HPAC, so the GCD profiles of the first and last five cycles in Figure 7f showed high symmetry, meaning that the overall resistance was not significant, even when increasing the thickness by 60% and after 30,000 cycles.

## 3. Materials and Methods

### 3.1. Chemicals

All reagents, including polyvinylpyrrolidone (PVP, (C_6_H_9_NO)_n_, average MW: 1,300,000, Sigma-Aldrich, St. Louis, MO, USA), potassium carbonate (anhydrous, K_2_CO_3_, 99%, Alfa Aesar, Heysham, UK), carbon black (Super P^®^, Timcal Ltd., Bodio, Switzerland), vapor-grown carbon nanofibers (VGCFs, 7 μm in length and 0.11 μm in diameter, Yonyu Applied Technology Material Co., Ltd., Tainan, Taiwan), and colloidal polytetrafluoroethylene (PTFE) dispersion (D1-E, Daikin Industries Ltd., Osaka, Japan), were adopted without further purification. Deionized (DI) water produced from a Milli-Q Integral water purification system (Millipore Ltd., Burlington, MA, USA) was utilized throughout the experiments.

### 3.2. Preparation of Hydrogel-Derived Hierarchical Porous Activated Carbon (H-HPAC) Encapsulated H-HPAC (H@H) Composite Material

To construct the H@H composite material, PVP powders and K_2_CO_3_ were well-dissolved in DI water individually. Here, the mass ratio between K_2_CO_3_ and PVP was controlled at 2 as no hydrogel is formed when the ratio was less than 2, as demonstrated in our previous study [9]. The concentrations of PVP and K_2_CO_3_ solutions were 14.3 wt.% and 40.7 wt.%, respectively. The volume ratio between PVP solution and K_2_CO_3_ solution was 2. Then, 0.5 g of H-HPAC was carefully added to the PVP solution, whereas the resultant was vigorously stirred to ensure homogenous mixing. Following the addition of K_2_CO_3_ solution dropwise to the PVP/H-HPAC solution, the black elastomer-like sample was completely obtained within 5 min (see Figure S1). The resulting composite hydrogel was dried in an oven at 120 °C for 12 h to completely evaporate the water molecules that were encapsulated within the matrix. The residues were then thermally pyrolyzed in a tube furnace at 900 °C for 2 h under an argon atmosphere with a flow rate of 200 mL/min, so the newly formed H-HPAC converted from the PVP would encapsulate the original H-HPAC. After repeated rinses with DI water, drying, and grinding procedures, the loose H@H powders can be obtained.

### 3.3. Characterizations

The crystalline structure of the as-prepared H@H composite material was identified using a powder X-ray diffractometer (XRD, D2 PHASER, Bruker AXS Inc., Karlsruhe, Germany) with a Cu target (λ = 1.541 Å) that was excited at 30 kV and 10 mA. The corresponding PXRD pattern was recorded in the range of 2*θ* from 10° to 70° at a scanning rate of 0.5 s/step. The Raman spectrum was collected between 1000 cm^−1^ and 1800 cm^−1^ by a confocal Raman microscope (inVia, Renishaw, UK) equipped with a 633 nm laser source. For morphological observations, the micrographs were acquired from the scanning electron microscope (SEM, JSM-IT200, JEOL Ltd., Tokyo, Japan) and a transmission electron microscope (TEM, JEM-2100, JEOL Ltd., Tokyo, Japan). To examine the textural properties, the N_2_ adsorption–desorption isotherm was measured at 77 K on a surface area and porosity analyzer (ASAP 2020 V3.00, Micromeritics Instrument Corporation, Norcross, GA, USA) after degassing under vacuum at 160 °C for 8 h. An elemental analyzer (FLASH 2000, Thermo Fisher Scientific Inc., Waltham, MA, USA) was applied for determining the percentages of carbon, nitrogen, and oxygen in the H@H composite material. The chemical environments were analyzed with X-ray photoelectron spectroscopy (XPS, PHI 5000 VersaProbe III, ULVAC-PHI, Inc., Kanagawa, Japan) with a beam size of 100 µm under Al K_α_ radiation (λ = 8.3406 Å). Their corresponding high-resolution spectra were further deconvoluted by the Gaussian–Lorentzian fitting method using an XPSPEAK 4.1 software.

### 3.4. Electrochemical Measurements

The electrochemical tests throughout this study were conducted in the symmetric two-electrode configuration at ambient conditions. To prepare the free-standing H@H electrodes, the ingredients (80 wt.% of H@H, 5 wt.% of Super P^®^, 5 wt.% of VGCFs, and 10 wt.% of PTFE) were mechanically blended and repeatedly calendared. The as-prepared H@H electrodes with a thickness of 160 ± 7 µm and a mass loading of 7.5 ± 0.8 mg/cm^2^ were obtained after drying at 130°C. To assemble the coin-type cells, 1 M TEABF_4_/PC and cellulose-based membrane (TF4535, NKK, Kochi, Japan) were used as the organic electrolyte and separator, respectively. The cyclic voltammograms (CVs) and electrochemical impedance spectroscopy measurements were recorded using a multichannel electrochemical workstation (VSP-3e, Bio-Logic, Seyssinet-Pariset, France). The electrochemical impedance spectra (EIS) were recorded at open circuit potential (OCP) from 100 kHz to 0.01 Hz with an AC potential amplitude of 5 mV. The galvanostatic charge–discharge (GCD) profiles and the cycling stabilities were evaluated through a computer-controlled system (CT-4008T-5V50mA, Neware Technology Limited, Shenzhen, China). To determine the specific capacitance (*C_s_*, F/g) of the H@H electrode in the symmetric supercapacitor, the value can be calculated from the GCD profiles by *C_s_* = 2 *It*/*mV*, where *I* is the applied current (A), *t* is the recorded discharge time (s), *m* is the mass of active material at one electrode (g), and *V* is the voltage window (volts). As for the energy density (*E*, Wh/kg) and power density (*P*, W/kg), they can be further acquired based on the equations *E* = *C_s_V*^2^/(2 × 4 × 3.6) and *P* = 3600 *E*/*t*, respectively [9,15].

## 4. Conclusions

In summary, this study presents an alternative concept for the construction of the hydrogel-derived HPAC (H-HPAC) encapsulated H-HPAC (H@H) composite material through the thermal pyrolysis of a PVP/H-HPAC hydrogel under an argon atmosphere at 900 °C. Compared to the original H-HPAC, the as-prepared H@H retains good purity, lower crystallinity, and high proportions of sp^2^-type carbons. However, H@H has a lower specific surface area and decreased pore parameters, but a substantial increase in the percentage of nitrogen species. Even with the notable change in the textural features, the symmetric supercapacitor assembled by the H@H electrode with a mass loading of 7.5 mg/cm^2^ and organic electrolyte still exhibits good reversible capacitance, comparable rate capability, and excellent cyclability. The results presented in this study support the H@H as a promising electrode material for other electrochemical energy storage fields, such as metal–ion capacitors.

## Figures and Tables

**Table 1:** Textural properties of H@H composite material and H-HPAC.

	Properties	SSA (m^2^/g)	V_t_^1^ (cm^3^/g)	V_ultra_^2^ (cm^3^/g)	V_micro_^3^ (cm^3^/g)	V_meso_^4^ (cm^3^/g)
Samples						
H@H		1316	0.62	0.21	0.51	0.11
H-HPAC ^5^		2012	1.16	0.11	0.69	0.47

^1^ V_t_: total pore (single-point) volume obtained from the amount of adsorbed nitrogen at P/P_0_ = 0.995. ^2^ V_ultra_: volume of ultramicropores (pores < 0.7 nm). ^3^ V_micro_: volume of micropores (pores < 2.0 nm). ^4^ V_meso_: volume of mesopores (difference between V_t_ and V_micro_). ^5^ The values were obtained from Ref. [9].

## Data Availability

Data sharing is not applicable to this article.

## Supplementary Materials

The following supporting information can be downloaded at: https://www.mdpi.com/article/10.3390/ijms23126774/s1.
