# Supplementary material for: Constructing a Carbon-Encapsulated Carbon Composite Material with Hierarchically Porous Architectures for Efficient Capacitive Storage in Organic Supercapacitors
Source: Int J Mol Sci. 2022 Jun 17;23(12):6774. doi: 10.3390/ijms23126774 (PMC9223422; doi:10.3390/ijms23126774)
Supplement: Supplementary file 1 [file ijms-23-06774-s001.zip › ijms-1759516-SM.pdf]

Supplementary Information

Constructing a Carbon-Encapsulated Carbon Composite Material with Hierarchically Porous Architectures for Efficient Capacitive Storage in Organic Supercapacitors

Rene Mary Amirtha [1,†], Hao-Huan Hsu [1,†], Mohamed M. Abdelaal [1,2], Ammaiyappan Anbunathan [1], Saad G. Mohamed [2], Chun-Chen Yang [1,3,4] and Tai-Feng Hung [1,*]

[1]Battery Research Center of Green Energy, Ming Chi University of Technology, 84 Gungjuan Rd., Taishan District, New Taipei City 24301, Taiwan

[2]Tabbin Institute for Metallurgical Studies (TIMS), Tabbin, Helwan 109, Cairo 11421, Egypt

[3]Department of Chemical Engineering, Ming Chi University of Technology, 84 Gungjuan Rd., Taishan District, New Taipei City 24301, Taiwan

[4]Department of Chemical and Materials Engineering, Chang Gung University, 259 Wenhua 1st Rd., Guishan District, Taoyuan 33302, Taiwan

[†]These authors contributed equally to this work

[*] Corresponding author.

E-mail addresses: taifeng@mail.mcut.edu.tw (T.F. Hung)

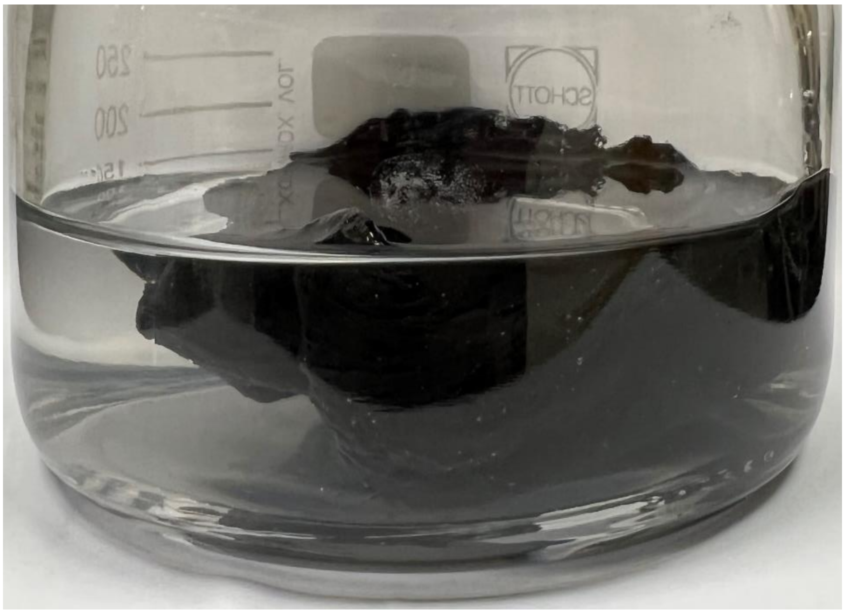

Figure S1. Digital image of PVP/H-HPAC composite hydrogel.

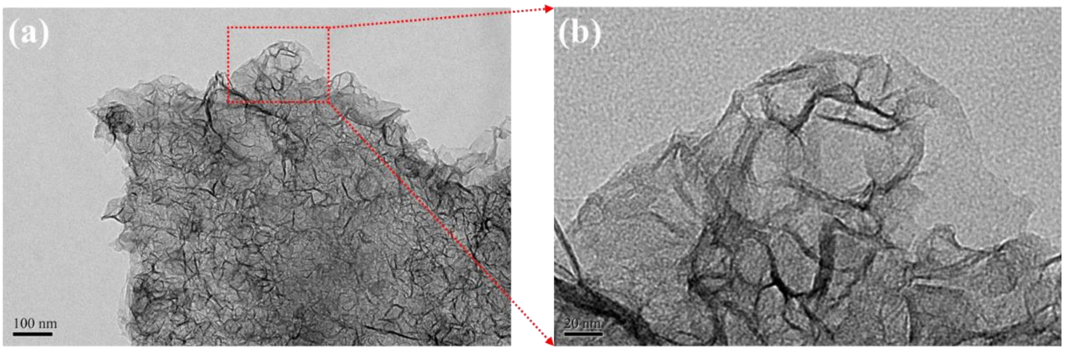

Figure S2. Low-magnification TEM micrographs of H@H composite material with the scale bar of (a) 100 nm and (b) 20 nm, respectively.

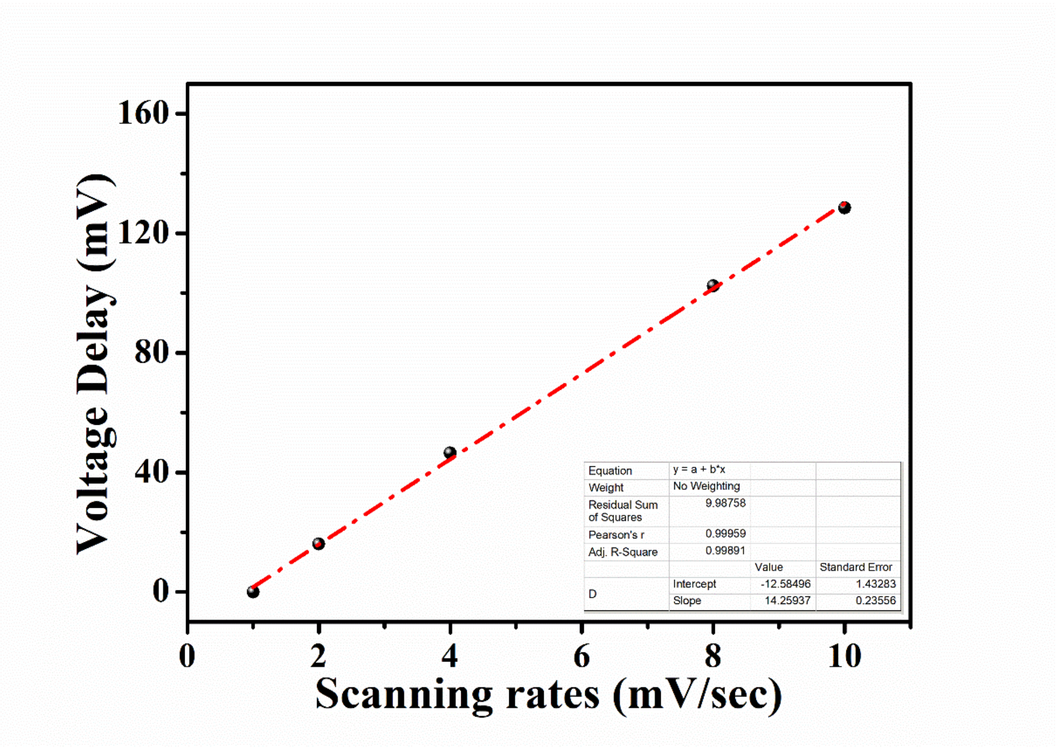

Figure S3. Dependence of the voltage delay (ΔV) on the scanning rates, in which the ΔV values were acquired from cyclic voltammograms plotted in Figure 6 (a).

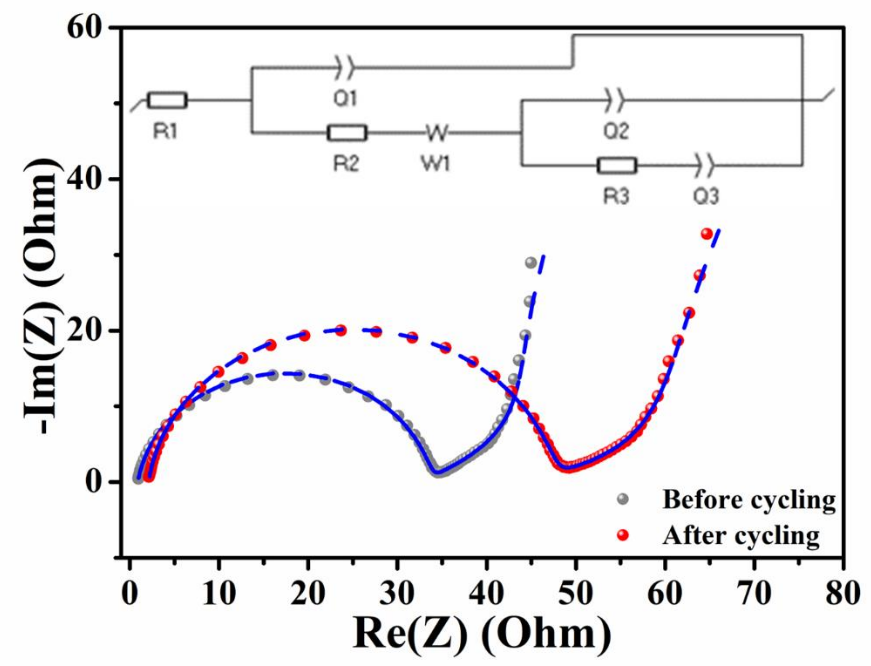

Figure S4. The electrochemical impedance spectra of the H@H electrode recording before and after the cycling test, while the inset shows the equivalent circuit model used for the parameter fitting.

Table S1. Comparison of electrochemical performances of H-HPAC and reported HPAC in symmetric supercapacitors using 1 M $TEABF_4$/PC electrolyte.

Heteroatoms	S_{BET} (m^2/g)	Mass loading (mg/cm^2)	Voltage (V)	Specific capacitance (F/g)	Capacitance retention (%)	References⁋
N (0.23 wt.%)<br>O (4.7 wt.%)	2012	5.1	0 ~ 2.7	118 at 1 mA/cm^2, 78 at 10 mA/cm^2	76 at 10,000 at 5 mA	9
O (~9 at. %)	1899	~0.8	0 ~ 2.7	134 at 0.5 A/g, 96 at 10 A/g	81 at 10,000 cycles at 2 A/g	15
N (4 at. %)<br>O (15.5 at. %)	1358	1.2	0 ~ 2.5	21 at 0.1 A/g, 11.5 at 5 A/g	92 at 5,000 cycles at 0.5 A/g	37
N (1.3 wt.%)<br>O (12 wt.%)	2125	NA†	0 ~ 2.5	139 at 0.25 A/g, 90 at 10 A/g	NA	38
N (~10.8 wt.%)	1519	~1.0	0 ~ 3.0	162 at 0.5 A/g, 103 at 10 A/g	~93 at 10,000 cycles at 2 A/g	39
S (NA)	2554	NA	0 ~ 2.7	173 at 0.05 A/g, 115 at 10 A/g	NA	40
O (NA)	1594‡	NA	0 ~ 2.5	88 at 0.5 A/g, 52 at 10 A/g	91 at 3,000 cycles at 5 A/g	41
N (1.1 wt. %)<br>O (4.6 wt. %)	2626	8.2	0 ~ 3.0	140 at 0.05 A/g, 92 at 10 A/g	~82 at 10,000 cycles at 2.5 A/g	42
N (0.67 wt.%)	2566	3.5	0 ~ 2.7	147 at 0.05 A/g, 117 at 10 A/g	81 at 10,000 cycles at 5 A/g	43
N (0.58 wt.%)<br>O (4.5 wt.%)	1316	7.5	0 ~ 2.7	119 at 1 mA/cm^2, 74 at 10 mA/cm^2	77 at 30,000 at 5 mA	This work

†Not available, ‡Based on QSDFT calculation, ⁋Same as those cited in Figure 7 (d).
